# Chamazulene Induces Metabolic Reprogramming and Mitigates Inflammation in Photoaged Skin: PPARα/γ as Potential Regulators

**DOI:** 10.3390/antiox14111320

**Published:** 2025-10-31

**Authors:** Ying Zhou, Wencui Wang, Lei He, Nan Zhang, Bowen Zhou, Zimeng Chen, Li Ma, Lei Yao

**Affiliations:** 1 Department of Resources and Environment, School of Agriculture and Biology, Shanghai Jiao Tong University, 800 Dongchuan Road, Shanghai 200240, China; zhouyingmap@sjtu.edu.cn; 2 Research and Development Center of Aromatic Plants, School of Design, Shanghai Jiao Tong University, 800 Dongchuan Road, Shanghai 200240, China; wwh123@sjtu.edu.cn (W.W.); fxzwzhangnan@sjtu.edu.cn (N.Z.); zhou.bw@sjtu.edu.cn (B.Z.); zimengchen@sjtu.edu.cn (Z.C.); 3 School of Public Health, Hongqiao International Institute of Medicine, Shanghai Jiao Tong University School of Medicine, 227 South Chongqing Road, Shanghai 200025, China; 018150910007@sjtu.edu.cn

**Keywords:** chamazulene, photoaging, PPARα/γ, metabolic reprograming, inflammation

## Abstract

Chamazulene (CHA) is a brilliant blue compound present in *Artemisia sieversiana* Ehrhart ex Willd. essential oil (AEO). We have previously reported that both CHA and AEO can shield the skin from UVB damage, exhibiting significant anti-photoaging effects. However, the molecular mechanisms underlying CHA’s photoprotective properties are still unclear. Herein, we integrated transcriptomics, targeted fatty acid profile, and untargeted metabolomics analyses on the dorsal skin of mice exposed to UVB with or without 0.4% CHA topical treatment. The results showed that CHA upregulated key genes involved in fatty acid metabolism, including two peroxisome proliferator-activated receptor (PPAR) subtypes, i.e., PPARα and PPARγ, in mouse skin. The CHA treatment elevated levels of various saturated, monounsaturated, and polyunsaturated fatty acids, and it especially restored n-3/n-6 polyunsaturated fatty acid homeostasis and downregulated the p38 MAPK/COX-2 pathway. Additionally, CHA enhanced skin non-essential amino acid metabolism, likely via PPARα. In conclusion, our study indicates that CHA may mitigate UVB-induced photoaging by inducing metabolic reprogramming and suppressing inflammation, and the findings suggest that the activation of PPARα/γ may play a vital role in these observed effects, thereby establishing CHA as a promising topical agent against UVB-induced photoaging.

## 1. Introduction

Skin aging is a universal and complex biological process driven by intrinsic factors, such as genetics, and extrinsic factors, including sun exposure, environmental pollution, and lifestyle [1,2]. Among these, ultraviolet (UV) radiation, particularly UVA and UVB rays, induces a number of lesions in the skin, leading to premature skin aging, a process known as photoaging [3]. Clinically, photoaging is characterized by various signs, including deep and coarse wrinkles, rough and leathery texture, pigmentation changes, and telangiectasias [4]. Over the past two decades, significant progress has been made in understanding the pathogenesis of photoaging. Briefly, photoaging is primarily driven by UV-induced reactive oxygen species (ROS) generation, which causes oxidative stress, DNA damage, and the activation of mitogen-activated protein kinase (MAPK). The MAPK signaling cascade leads to AP-1 heterodimerization formation, which successively increases the expression of matrix metalloproteinases (MMPs). This process results in the degradation of extracellular matrix (ECM) components, e.g., collagen and elastin [5]. Simultaneously, UV radiation activates inflammatory responses in the skin. The nuclear factor-κB (NF-κB) and p38 MAPK pathways, as well as the inflammasome, play key roles in this process by provoking a strong increase in the secretion of cytokines and chemokines, including interleukins (ILs), and CC and CXC chemokine ligands (CCLs and CXCLs) [6]. While genomic and proteomic signatures of photoaging have been extensively characterized, emerging evidence suggests that UV-induced alterations in glucose, nicotinamide, glutathione, and lipid metabolism contribute to skin dysfunction, particularly through elevated oxidative stress, inflammation, and collagen destruction [7,8,9,10]. Of these metabolic changes, fatty acid dysregulation is particularly noteworthy, as fatty acids are recognized not only for their well-established roles in energy supply and biological membrane composition but also as vital regulators of skin barrier function and inflammation [11,12]. Notably, photoaged human skin exhibits remarkable downregulation of fatty acid biosynthesis [8,13], indicating that fatty acid dysregulation is a hallmark of photoaging.

The peroxisome proliferator-activated receptors (PPARs) are ligand-activated nuclear receptors involved in the transcriptional regulation of energy balance, e.g., lipid/fatty acid and glucose metabolism [14]. However, PPARs have other important functions beyond their canonical metabolic roles, including regulation of cell proliferation, differentiation, inflammatory response, and barrier homeostasis in the skin, thus representing potential targets for understanding and treating various skin diseases and disorders [15]. To date, three PPAR isoforms have been identified, namely, PPARα, PPARβ/δ, and PPARγ. Crucially, UV radiation has been shown to suppress the expression of PPARs [8,16]. Deficiency in PPARγ has been linked to skin cell apoptosis, inflammation, and barrier dysfunction upon UVB exposure [17]. These findings open new perspectives in targeting PPARs as a potential novel approach for the treatment of photoaging. Recent studies have identified that several synthetic or plant-derived PPAR agonists decrease ROS generation, suppress the expression of MMPs, promote the synthesis of collagen, and inhibit the production of proinflammatory cytokines [18,19,20,21,22,23]. However, whether these PPAR agonists can counteract photoaging by restoring UV-induced disruptions in cutaneous metabolism and barrier homeostasis remains largely unknown.

Despite being an FDA-approved topical drug for treating photoaging, tretinoin’s clinical use is often limited by side effects such as burning, scaling, and dermatitis [24]. As a result, those natural phytochemicals with fewer adverse effects have gained increasing attention, e.g., flavonoids, phenolics, and essential oil components [25]. Chamazulene (1,4-dimethyl-7-ethylazulene, CHA) is a bicyclic sesquiterpene with a vibrant blue color. It is produced at high temperatures during the hydrodistillation of essential oils from certain Asteraceae plants, such as German chamomile, wormwood, and yarrow [26]. In addition to its use as a natural coloring agent in cosmetics, CHA has shown remarkable efficacy in alleviating inflammation and smoothing skin [26,27]. In our previous study, CHA was shown to mitigate UVB-induced damage in HaCaT cells, including the depletion of the endogenous antioxidant system, mitochondrial depolarization, and the production of proinflammatory markers [28]. Additionally, a topical emulsion of *Artemisia sieversiana* Ehrhart ex Willd. essential oil (AEO), which is rich in CHA, exerted significant anti-photoaging effects on UVB-irradiated mouse skin [29]. These findings collectively highlight the potential of CHA as a promising candidate for shielding the skin from photoaging, and a comprehensive exploration of the underlying mechanisms is required.

The purpose of this study is to characterize the metabolomic reprogramming associated with topical CHA application in UVB-irradiated mouse skin by employing multi-omics approaches. Firstly, we utilized RNA sequencing analysis to identify the specific alterations in the transcriptome of UVB-exposed mouse skin with CHA treatment. The results showed that CHA’s photoprotective effects may be linked to activation of PPARα/γ, which occupies a central hub in enhancing fatty acid metabolism and reducing inflammation in UVB-exposed mouse skin with CHA treatment. Secondly, to establish a causative role for PPARα/γ activation in mitigating photoaging, targeted fatty acid profiling and untargeted metabolomics analyses of mouse skin were performed. The data demonstrated that CHA improved fatty acid metabolism, especially polyunsaturated fatty acid (PUFA) homeostasis, and upregulated non-essential amino acid (NEAA) metabolism in UVB-irradiated mouse skin. These findings, for the first time, underscore the vital role of CHA in resisting photoaging through PPARα/γ activation, highlighting its substantial potential for cosmetic or pharmaceutical applications.

## 2. Materials and Methods

### 2.1. A 0.4% CHA Formulation Preparation

Chamazulene (CHA) was isolated from AEO, as detailed in our previous work [28]. Since our initial study demonstrated that an oil-in-water (O/W) emulsion fortified with 1.5% AEO showed no toxicity but significantly attenuated photoaging [29], we incorporated CHA into the same emulsion system at a final concentration of 0.4% (*w*/*w*) in the current study, representing a proportional bioactive-equivalent concentration (Figure S1). This concentration was selected based on the assumption that the essential oil contains approximately 38.92% CHA. This adjustment ensures that the dose is consistent with previous studies while accounting for the concentration of the active compound.

### 2.2. Animal Experiment and Treatment

A total of eighteen female ICR mice (8 weeks old) weighing between 18 and 22 g were obtained from Shanghai Slac Laboratory Animal Co. Ltd. (Shanghai, China) and housed in Shanghai Jiao Tong University Laboratory Animal Center under controlled temperature and light conditions (23 ± 2 °C, 12 h light–dark cycle). The manipulation of animals and experimental procedures were all approved by the Institutional Animal Care and Use Committee of Shanghai Jiao Tong University (A2023092). The mice were randomly divided into three groups, with six mice in each group as follows: normal control group (NC), model control group (MC), and 0.4% CHA-treated group (CHA). After one week of adaptive feeding, the dorsal skin (2 cm × 3 cm) of the mice was depilated. Mice in the NC group had neither UV radiation nor drug administration. Mice in the MC group were merely exposed to UVB radiation with an irradiation device equipped with a TL20W/01 UVB lamp (Philips, Amsterdam, The Netherlands). The irradiation lasted for 5 weeks, and the total dose was 14.877 J/cm^2^ per mouse. The specific UVB irradiation procedure can be found in our previous work [29]. Mice in the CHA group received a topical application of a 0.4% CHA-containing emulsion at a dose of 0.22–0.24 g per mouse. Fifteen minutes later, mice were exposed to UVB irradiation. Twenty-four hours after irradiation completion, mice were euthanized, and the dorsal skin was excised and promptly rinsed with ice-cold PBS. One skin fragment was fixed in 4% paraformaldehyde and embedded in paraffin for sectioning. The remaining fragments were frozen at −80 °C for transcriptome sequencing, metabolomics analysis, and Western blotting.

### 2.3. Histopathological Analysis

Paraffin-embedded sections were stained with hematoxylin and eosin (H&E) for general histopathology and to measure epidermal thickness. The collagenous fiber and elastic fiber in the dermis were detected using Masson’s trichrome and Gomori staining, respectively.

### 2.4. Determination of Hydroxyproline

Mouse skin samples (*n* = 6 per group) were subjected to acidic hydrolysis to break down collagen into its amino acids. The hydroxyproline (HYP) content in the hydrolysate, a marker usually used to quantify collagen [30], was determined through a commercial colorimetric assay kit (Sangon, Shanghai, China).

### 2.5. RNA Sequencing Analysis

Mouse skin samples (*n* = 3 for each group) were ground at low temperature, and total RNA was extracted using TRIzol reagent (Invitrogen, Carlsbad, CA, USA). RNA concentration and purity were determined using a NanoDrop 2000 spectrophotometer (Thermo Fisher Scientific, Waltham, MA, USA). RNA integrity was evaluated by the 2100 Bioanalyzer system (Agilent, Santa Clara, CA, USA). Approximately 1 μg of total RNA of each sample was employed to prepare the transcriptome sequencing library using a VAHTS Universal V6 RNA-seq Library Prep Kit for Illumina (Vazyme Biotech, Nanjing, China). The libraries were sequenced on an Illumina Novaseq 6000 platform (Shanghai Oebiotech Co., Ltd., Shanghai, China), and 150 bp paired-end reads were generated. With low-quality reads removed from raw reads, clean reads were aligned to the mouse genome using the HISAT2 software (v 2.1.0). HTSeq-count was used to acquire read counts of each gene, and fragment per kilobase of transcripts per million mapped reads (FPKMs) were calculated to evaluate the gene expression value. Principal component analysis (PCA) was conducted to assess overall data variation in the transcriptome data. The genes were identified as differentially expressed genes (DEGs) using the DESeq 2 algorithm based on a significant threshold at a *q*-value < 0.05 and fold change > 1.5 or <0.67, followed by Kyoto Encyclopedia of Genes and Genomes (KEGG) functional enrichment analysis.

### 2.6. Untargeted Metabolomic Analysis

#### 2.6.1. Sample Preparation and Ultra-Performance Liquid Chromatography–Tandem Mass Spectrometric (UPLC-MS/MS) Analysis

About 30 mg skin samples from each mouse (*n* = 6 for each group) were homogenized in 400 μL of methanol–water (4:1, *v*/*v*) containing an internal standard mixture (4 μg/mL). The internal standard mixture consisted of L-2-chlorophenylalanine, succinic acid-d4, L-valine-d8, and cholic acid-d4. After vortexing and an ice bath, the samples were centrifuged (12,000 rpm, 4 °C, 10 min). An aliquot of 300 μL of the supernatant was transferred and evaporated under a nitrogen stream. The resulting residue was reconstituted into 300 μL methanol–water (1:4, *v*/*v*) and filtered through a 0.22 μm membrane for UPLC-MS/MS analysis.

An ACQUITY UPLC I-Class plus (Waters, Milford, MA, USA) coupled with an ACQUITY UPLC HSS T3 column (100 mm × 2.1 mm, 1.8 μm; Waters, Milford, MA, USA) was used for the separation of metabolites. The column temperature was maintained at 45 °C. The column flow rate was 0.35 mL/min, and the injection volume was 3 μL. The mobile phases consisted of 0.1% formic acid in water (A) and acetonitrile (B). The gradient elution program for both positive and negative ion mode was as follows: 0–4 min, 5% B; 4–8 min, 5–30% B; 8–10 min, 30–50% B, 10–14 min, 50–80% B; 14–15 min, 80–100% B, and 15–16 min, 5% B. A Q Extractive Plus mass spectrometer (Thermo Fisher Scientific, Waltham, MA, USA) with an ESI source was used to identify metabolites. The parameters were set as follows: sheath gas flow rate, 35 Arb; auxiliary gas flow rate, 8 Arb; spray voltage, −3 kV for the negative mode and 3.8 kV for the positive mode; capillary temperature, 320 °C. Data was acquired using the full MS-ddMS2 setup with a mass range of 70–1050 *m*/*z*.

#### 2.6.2. Data Processing and Multivariate Analysis

The raw data generated by UPLC-MS/MS was processed with the Progenesis QI v3.0 software (Nonlinear Dynamics, Newcastle, UK), which performed peak recognition, peak alignment, and peak area normalization. Identification of metabolites was conducted by comparison of their retention times (RTs), exact masses, product ions, isotopes, and MS/MS fragments with publicly available databases (Human Metabolome Database, Lipidmaps v 2.3, and METLIN) and an in-house standard library (LuMet-Animal 3.0). The identification levels were established as follows: Level 1 refers to confidently identified metabolites (i.e., matching their retention time and accurate mass with the standards and MS/MS fragment score > 45). Level 2 corresponds to putatively annotated compounds (i.e., matching their retention time and accurate mass with the standards and MS/MS fragment score < 45). Level 3 includes a class of putatively characterized compounds (i.e., comparing the accurate mass to available databases and MS/MS fragment score > 45). Level 4 covers all remaining unknown metabolites. The normalized data were subjected to multivariate statistical analysis. Specifically, principal component analysis (PCA) and orthogonal partial least squares-discriminant analysis (OPLS-DA) were performed using R (v 3.2.0) to evaluate the differences among three experimental groups. Based on the variable importance in the projection (VIP) obtained from OPLS-DA analysis and the *p*-value of Student’s *t*-test, the differentially expressed metabolites (DEMs) were identified with a VIP value > 1 and *p*-value < 0.05.

### 2.7. Determination of Fatty Acid Composition by Gas Chromatography–Mass Spectrometry (GC-MS)

Approximately 50 mg of skin tissue from each mouse (*n* = 6 per group) was transferred into a 10 mL glass tube, and 5 mL of cold dichloromethane/methanol (2:1 *v*/*v*) was added. The mixture was thoroughly vortexed and subjected to ultrasonication for 30 min at a low temperature. Then, 2 mL of water was added, and the organic phase (lower layer) containing extracted fatty acids was collected and dried under a stream of nitrogen. The dried samples were redissolved in 2 mL of n-hexane, spiked with 50 μg of internal standard (nonadecanoic acid methyl ester, Nu-Chek Prep, Elysian, MN, USA), and subsequently mixed with 2 mL of sulfuric acid–methanol solution (1:10 *v*/*v*). The mixture was incubated at 80 °C for 30 min to generate fatty acid methyl esters (FAMEs), according to the method described by Zhou et al. [31]. Then, 2 mL of water was added, and the upper organic phase was collected and dried under a nitrogen stream. The FAMEs were finally reconstituted in 1 mL of n-hexane for GC-MS analysis.

Each sample (1 μL) was injected into a 7890B gas chromatograph coupled with a 5977B mass selective detector (Agilent, Santa Clara, CA, USA) operating in positive ionization mode. The FAMEs were separated on a DB-23 column (30 mm × 0.25 mm, 0.25 mm, Agilent, Santa Clara, CA, USA) under the following temperature program: the initial column temperature was set at 140 °C, ramped up at a rate of 5 °C/min up to 300 °C, and maintained for 10 min. Helium gas was used as the carrier gas at a flow rate of 1.8 mL/min. Mass spectrometry data were obtained in single ion monitoring (SIM) mode. Peaks were analyzed using the Agilent MSD ChemStation software (v B.04.03) and identified by comparing their retention time with that of pure FAME standards (Nu-Chek Prep, Elysian, MN, USA). Quantification of fatty acids was performed based on the linear curve of each standard reference (Table S4).

### 2.8. Quantitative Real-Time PCR (qRT-PCR) Analysis

Total RNA was extracted from mouse skin samples using TRIzol reagent, followed by cDNA synthesis using a HiScript II Q Select RT SuperMix kit (Vazyme Biotech, Nanjing, China). qRT-PCR was performed using a ChamQ Universal SYBR qPCR Master Mix kit (Vazyme Biotech, Nanjing, China), with GAPDH as the endogenous reference. All experiments were performed in triplicate. The primers used are noted in Table S1.

### 2.9. Western Blotting

Mouse skin proteins were extracted with ice-cold RIPA buffer containing protease inhibitor and protein phosphatase inhibitor and quantified using a BCA protein assay kit. Briefly, 30 μg of protein samples were loaded and separated with 10% SDS-PAGE and then transferred onto PVDF membranes. The membranes were blocked in 5% skim milk powder for 30 min at room temperature and then incubated overnight at 4 °C with specific primary antibodies. The primary antibodies used in this study were PPARα (1:1000, Affinity Biosciences, Changzhou, China), PPARγ (1:1000, Proteintech, Wuhan, China), COX-2 (1:1000, Proteintech, Wuhan, China), p38 MAPK (1:2000, Proteintech, Wuhan, China), and p-p38 MAPK (1:1000, Proteintech, Wuhan, China). GAPDH (1:5000, Huabio, Hangzhou, China) was used as the loading control. After washing with TBST, membranes were incubated with horseradish peroxidase (HRP)-conjugated goat anti-rabbit secondary antibody (1:3000; Jackson ImmunoResearch, West Grove, PA, USA) for 1 h at room temperature. Protein bands were visualized by an ECL assay kit on a Tanon 4800 Chemiluminescent Imaging System (Tanon, Shanghai, China) and quantified using the ImageJ software (v 1.54i).

### 2.10. Statistical Analysis

Data were analyzed using the Origin 2023b software and expressed as mean ± SD (standard deviation). Statistical comparisons across multiple groups were performed using one-way ANOVA analysis followed by Tukey’s post hoc test, whereas comparisons between two groups were conducted using an unpaired *t*-test. *p*-values < 0.05 were considered to be statistically significant.

## 3. Results

### 3.1. CHA Exhibited Anti-Photoaging Effect by Mitigating UVB-Induced Damage in Mouse Skin

**Figure 1:**
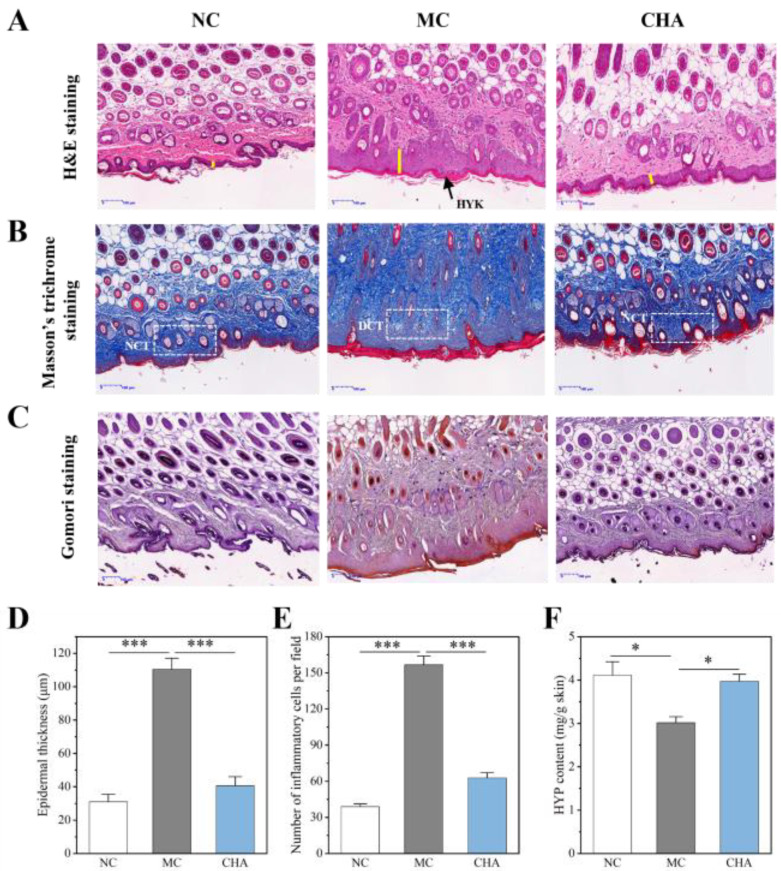
Evaluation of the anti-photoaging effects of CHA with histopathological analysis. Histological staining was performed on serial sections of the dorsal skin of each group of mice. Representative images are shown at 50× magnification (scale bars = 100 μm). (**A**) H&E staining. The yellow lines show the epidermis layer of the skin. HYK: Hyperkeratosis. (**B**) Masson’s trichrome staining. Blue staining indicates collagen fibers. DCT: damaged collagen fibers; NCT: normal collagen fibers. (**C**) Gomori staining. Purple staining indicates the elastic fibers. (**D**) Epidermal thickness and (**E**) number of inflammatory cells were quantified from high-power field images of six replicates of each group (five measurements per image). (**F**) Hydroxyproline (HYP) assay on mouse skin. All values are presented as mean ± SD (*n* = 6 in each group), * *p* < 0.05, *** *p* < 0.001, compared to the MC group.

In order to evaluate the histological alterations in UVB-exposed mouse skin with topical CHA application, representative images of H&E, Masson’s trichrome, and Gomori staining are presented in Figure 1A–C. H&E staining showed that UVB exposure caused direct epidermal damage associated with photoaging, including hyperkeratosis, epidermal thickening, aberrant dermal–epidermal junction, and inflammatory cell infiltration. Conversely, treatment with CHA alleviated this damage; the epidermal structure of mice in the CHA group closely resembled that of the NC group, with significant reductions in epidermal thickness and the number of inflammatory cells compared to the MC group (Figure 1D,E and Figure S2). Masson’s trichrome and Gomori staining were used to visualize alterations in dermal collagen and elastic fibers, respectively. CHA effectively prevented UVB-induced loss of collagen and elastic fibers, as evidenced by the abundant and dense distribution of these two ECM components in the NC and CHA groups compared with the MC group. Additionally, the HYP content was restored to normal levels following CHA treatment (Figure 1F). These findings suggested that CHA can enhance skin structure remodeling and suppress inflammation, offering a novel therapeutic approach to address skin damage associated with photoaging.

### 3.2. CHA Induced Transcriptomic Changes in the Skin of Photoaged Mouse Model

**Figure 2:**
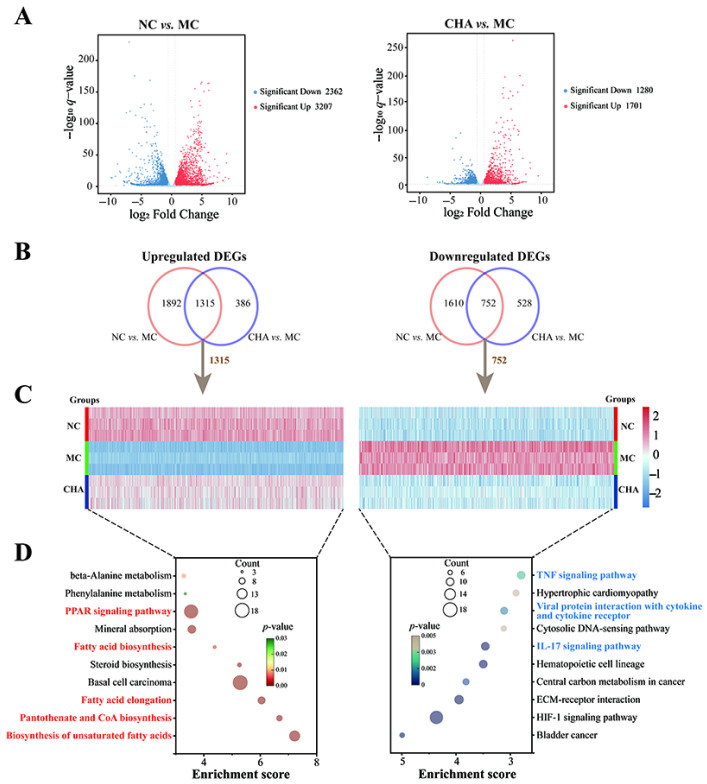
Transcriptional effects of CHA on the skin of a photoaged mouse model. (**A**) Volcano plots showing DEGs (*q*-value < 0.05 and |log2fold change| > 0.58) identified in NC vs. MC (left) and CHA vs. MC (right) comparisons. Red and blue dots represent significantly up- and downregulated genes, respectively. (**B**) Venn diagrams showing overlapping of DEGs between the two comparisons: upregulated (left), downregulated (right). (**C**) Heatmap visualization for overlapped DEGs between the two comparisons: upregulated (left), downregulated (right). Each column represents the column-normalized expression pattern of a single gene. Red indicates upregulation. Blue indicates downregulation. (**D**) KEGG pathway enrichment analysis for overlapped DEGs between the two comparisons: upregulated (left), downregulated (right). Data were obtained from three independent biological replicates.

To probe the molecular events clarifying the effects of topical CHA treatment, we performed RNA sequencing (RNA-seq) analysis on skin samples from NC-, MC-, and CHA-treated mice. According to the principal components analysis (PCA) of the skin transcriptome data, notable differences were found among these three groups (Figure S3). Volcano plots depict the identified DEGs in NC vs. MC and CHA vs. MC, and the numbers were 5569 and 2981, respectively (Figure 2A). Venn diagrams illustrate that the NC and CHA groups had 2067 genes in common, with 1315 upregulated and 752 downregulated as compared to the MC group (Figure 2B,C). These shared genes accounted for 69.3% of total DEGs in CHA vs. MC, possibly playing a crucial role in the anti-photoaging effects of CHA. Therefore, separate KEGG functional enrichment analysis for the up- and downregulated DEGs was conducted to identify the biological processes and signaling pathways regulated by CHA (Figure 2D). The upregulated DEGs were significantly enriched in pathways related to fatty acid metabolic process (e.g., biosynthesis of unsaturated fatty acids, pantothenate and CoA biosynthesis, fatty acid elongation, fatty acid biosynthesis, and the PPAR signaling pathway), while downregulated DEGs were linked to inflammatory responses (e.g., the IL-17 signaling pathway, viral protein interaction with cytokines and cytokine receptors, and the TNF signaling pathway). These findings strongly implied that CHA exerted anti-photoaging effects by enhancing fatty acid metabolism and attenuating inflammatory responses.

### 3.3. CHA Treatment Enhanced Fatty Acid Metabolism in the Skin of Photoaged Mouse Model, Potentially Involving PPARα/γ Activation

**Figure 3:**
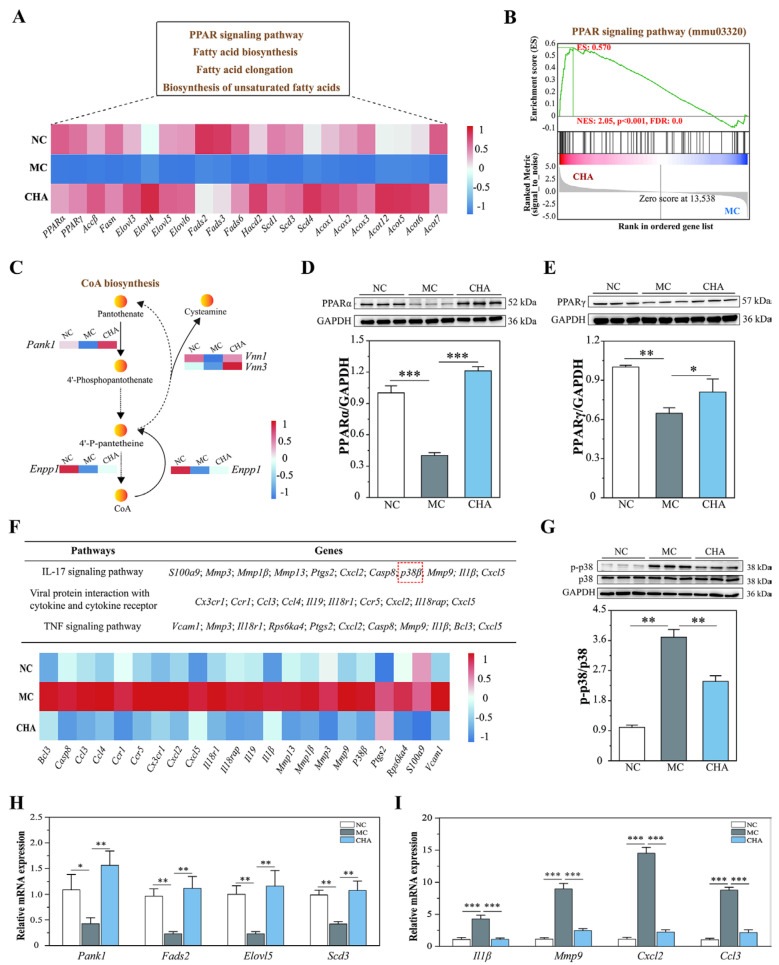
Role of PPARα/γ signaling in CHA-enhanced fatty acid metabolism and reduced inflammation in the skin of a photoaged mouse model. (**A**) Heatmap showing represented DEGs involved in fatty acid biosynthesis and β-oxidation according to the KEGG pathways. Each column represents the mean expression of a single gene from three independent biological replicates per condition (*n* = 3). Red represents a higher level of gene expression. Blue represents a lower level of gene expression. (**B**) Gene set enrichment analysis (GSEA) plot for the PPAR signaling pathway (CHA vs. MC). ES: enrichment score. NES: normalized enrichment score. FDR: false discovery rate. (**C**) DEGs involved in the CoA biosynthesis pathway. (**D**,**E**) Western blot analysis of PPARα and PPARγ protein expression in the mouse skin from NC, MC, and CHA groups. The results are normalized to GAPDH and shown as mean ± SD (*n* = 3). (**F**) Heatmap of downregulated DEGs related to skin inflammation according to the KEGG pathways. (**G**) Western blot analysis of p38 MAPK activity in mouse skin from NC, MC, and CHA groups. Quantification data (normalized to p38 MAPK) are presented as mean ± SD (*n* = 3). (**H**,**I**) Validation of DEGs by qRT-PCR. Gene expression levels were quantified using qRT-PCR and normalized to *GAPDH*. Data are presented as mean ± SD from three independent experiments (*n* = 3). * *p* < 0.05, ** *p* < 0.01, *** *p* < 0.001, compared to the MC group.

In this study, we identified a subset of genes encoding enzymes associated with fatty acid biosynthesis, including *Accβ*, *Fasn*, *Elovl3*, *Elovl4*, *Elovl5*, *Elovl6*, *Fads2*, *Fads3*, *Fads6*, *Hacd2*, *Scd1*, *Scd3*, and *Scd4*, as well as those engaged in fatty acid β-oxidation, such as *Acox1*, *Acox2*, *Acox3*, *Acot12*, *Acot5*, *Acot6*, and *Acot7*. As shown in Figure 3A, these genes were consistently downregulated in the MC group but upregulated in the CHA group. These findings highlight the importance of the PPAR signaling pathway, as PPARs are important transcriptional regulators controlling fatty acid metabolism. Specifically, PPARα primarily regulates fatty acid β-oxidation through modulating the expression of ACOX and ACOT [32,33], while PPARγ participates in fatty acid biosynthesis by directly or indirectly regulating ACC, ELOVL, SCD, and FASN [34,35,36,37]. In keeping with these DEGs involved in fatty acid metabolic processes, we observed that CHA restored the expression of *Pparα* and *Pparγ* and significantly upregulated the PPAR signaling pathway through a gene set enrichment analysis (GSEA) (Figure 3B). In addition, specific attention was given to the DEGs involved in the CoA biosynthesis pathway, as CoA serves as an acyl-group carrier in both fatty acid biosynthesis and β-oxidation [38]. The first step of CoA biosynthesis is the phosphorylation of pantothenate into 4’-phosphopantothenate by PANK, a rate-limiting enzyme that can be positively regulated by PPARα [39]. Herein, CHA treatment significantly upregulated the expression of *Pank1*, *Vnn1*, *Vnn3*, and *Enpp1*, which were suppressed in the MC group (Figure 3C), possibly reflecting the upregulation of fatty acid metabolism in the skin of CHA-treated mice. The effects of CHA on PPARα and PPARγ protein expression were subsequently assessed by Western blot analysis. The results showed that CHA significantly increased PPARα and PPARγ protein levels, which were reduced in UVB-irradiated skin (Figure 3D,E). Furthermore, the differential expression of randomly selected genes (*Pank1*, *Fads2*, *Elovl5*, and *Scd3*) was validated by qRT-PCR analysis (Figure 3H), and the results were consistent with the results of RNA-Seq. These findings strongly suggest that the activation of PPARα/γ is a critical mechanism responsible for the enhanced fatty acid metabolism in CHA-treated mouse skin.

### 3.4. CHA Treatment Blocked p38 MAPK Signaling in the Skin of Photoaged Mouse Model

In order to identify the key signaling molecule that is responsible for UVB-induced inflammation, the downregulated pathways were scrutinized. As shown in Figure 3F, pro-inflammatory genes involved in photoaging, such as *Ccl3*, *Ccl4*, *Cxcl2*, *Cxcl3*, *Cxcl5*, *Il19*, *Il1β*, *Mmp1β*, *Mmp3*, *Mmp9*, *Mmp13*, and *Ptgs2*, were significantly downregulated by CHA treatment. We validated the expression of *Il1β*, *Mmp9*, *Cxcl2*, and *Ccl-3* using qRT-PCR (Figure 3I), and the results were consistent with the RNA-seq data. Within the IL-17 signaling pathway, we found that CHA suppressed the expression of *p38β* (Figure 3F and Figure S4), a critical signaling molecule belonging to the MAPK family. It is well established that p38 MAPK is activated upon exposure to UV radiation, leading to the expression of various inflammatory mediators in the skin. Therefore, the impact of CHA on p38 MAPK activity (protein phosphorylation) was further investigated via Western blotting. The results showed enhanced phosphorylation of p38 MAPK in the MC group, but no significant elevation was found in the CHA group (versus the NC group) (Figure 3G). Therefore, CHA treatment effectively blocks p38 MAPK signaling, a crucial pathway involved in UVB-induced inflammation in the skin.

### 3.5. CHA Treatment Stimulated Metabolic Reprogramming in the Skin of Photoaged Mouse Model

To further clarify the activating effect of CHA on PPARα/γ, we proceeded to quantify the fatty acids in the mouse skin of these three groups, and the results are presented in Table 1 and Table S2. Strikingly, the activating effects of CHA on PPARα/γ resulted in the upregulation of multiple saturated, monounsaturated, and polyunsaturated fatty acids in the photoaged skin. The most abundant fatty acids included myristic acid (C14:0), palmitic acid (C16:0), palmitoleic acid (C16:1n-7), oleic acid (C18:1n-9), linoleic acid (C18:2n-6), and α-linolenic acid (C18:3n-3). As expected, levels of these fatty acids, as well as total saturated fatty acids (SFAs), total monounsaturated fatty acids (MUFAs), and total polyunsaturated fatty acids (PUFAs), were significantly reduced in UVB-irradiated skin, but CHA treatment restored their levels.

**Figure 4:**
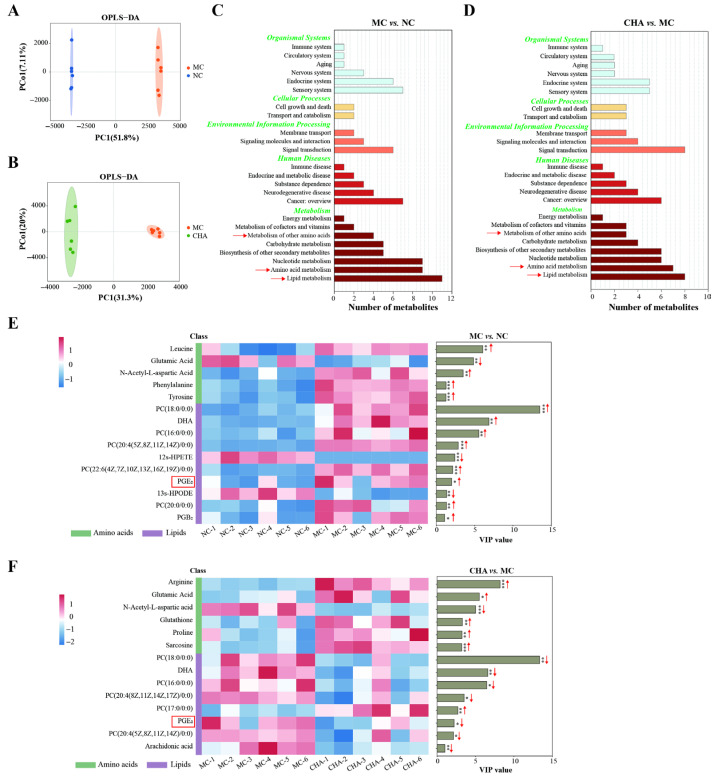
Effects of CHA on skin metabolite profiles of a photoaged mouse model. (**A**,**B**) OPLS-DA score plots of NC vs. MC and CHA vs. MC comparisons. Data obtained in the positive and negative ionization modes in the UPLC-MS/MS of each group were combined for OPLS-DA. Model predictability and statistical validity were evaluated using a permutation test, and the parameters are shown in Figure S5. The obtained R2Y value and Q2 value demonstrate the goodness of fit and predictability of each model, respectively. Data are obtained from six independent biological replicates. (**C**,**D**) Function annotation of unambiguously identified DEMs in NC vs. MC and CHA vs. MC comparisons based on KEGG pathway enrichment analysis. The corresponding categories at Level 1 are labeled in green. Differential functional pathways at Level 2 are labeled in black. (**E**,**F**) Visualization of differential lipids and amino acids in NC vs. MC and CHA vs. MC comparisons. These metabolites were identified through ion annotation and further confirmed by standards. The heatmaps display the Z-scored-normalized abundance of differential metabolites. Red reflects a higher level of abundance, and blue reflects a lower level of abundance. The VIP score is obtained from OPLS-DA analysis. * *p* < 0.05, ** *p* < 0.01, *** *p* < 0.001.

The impacts of PPARα/γ activation may not be limited to fatty acid metabolism, considering previous published work reported PPARα’s regulation of other metabolic processes in vivo, e.g., amino acid metabolism [40,41,42]. For this reason, we further characterized the metabolic alterations in mouse skin induced by UVB exposure and the effects of CHA treatment through untargeted metabolomic analysis. The orthogonal partial least squares discriminant analysis (OPLS-DA) model, a supervised method that allows for discrimination between two groups, revealed significant differences in metabolic profiles between UVB-exposed skin and the NC group. Importantly, CHA treatment effectively improved the UVB-induced metabolic alterations (Figure 4A,B). Moreover, KEGG pathway analysis of unambiguously identified metabolites (VIP value > 1 and *p*-value < 0.05) indicated that the differences observed in the two comparisons were primarily attributed to the changes in metabolism (Level 1), with the most significantly altered pathways (Level 2) identified as lipid metabolism and amino acid metabolism (Figure 4C,D). The key differentially expressed lipids and amino acids were further screened out, as illustrated in Figure 4E,F. Our observations indicated that most detected lipids were increased in the UVB-irradiated group, such as PGE_2_, DHA, PC(18:0/0:0), PC(16:0/0:0), and PC(20:4(5Z,8Z,11Z,14Z)/0:0). In contrast, CHA treatment resulted in the decline of their levels. UVB radiation also significantly increased various amino acids, including tyrosine, phenylalanine, N-acetyl-L-aspartic acid, and leucine, while a decline in the glutamic acid level was noted in the MC group. CHA treatment partially counteracted these changes by restoring N-acetyl-L-aspartic acid and glutamic acid. Most importantly, the levels of arginine, glutathione, proline, and sarcosine were upregulated by CHA treatment as compared to the irradiated group.

### 3.6. CHA Treatment Upregulated Non-Essential Amino Acid Metabolism in the Skin of Photoaged Mouse Model

**Figure 5:**
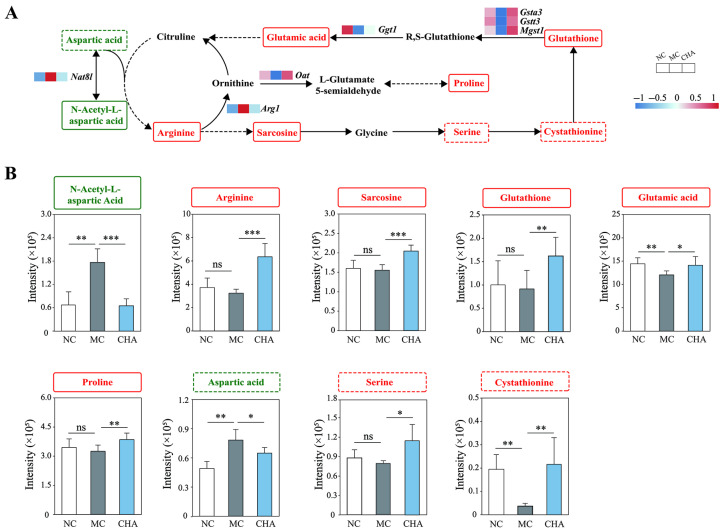
Promoting effects of CHA on non-essential amino acid (NEAA) metabolism in the skin of a photoaged mouse model. (**A**) Schematic representation of the NEAA metabolism pathway in CHA-treated mouse skin. Metabolites labeled in red and green were detected by UPLC-MS/MS. Red indicates upregulation, while green indicates downregulation in the CHA group compared to the MC group. Arrows indicate enzymatic conversions within the pathway. (**B**) The relative intensity of indicated NEAAs is presented as mean ± SD (*n* = 6). * *p* < 0.05, ** *p* < 0.01, *** *p* < 0.001, compared to the MC group. ns indicates no significant difference when compared to the MC group. N-acetyl-L-aspartic acid, arginine, sarcosine, glutathione, glutamic acid, and proline were identified as Level 1 (i.e., matching their retention time and accurate mass with the standards and MS/MS fragment score > 45); aspartic acid, serine, and cystathionine were identified as Level 2 (i.e., matching their retention time and accurate mass with the standards and MS/MS fragment score < 45). The detailed identification of N-acetyl-L-aspartic acid, arginine, sarcosine, glutathione, glutamic acid, and proline can be seen in Table S3.

Traditionally, amino acids have been classified as nutritionally essential or non-essential for humans and other mammals. Essential AAs (EAAs) are defined as those that cannot be synthesized de novo or are insufficiently synthesized relative to metabolic needs by organisms and thus must be obtained from foods or supplements; conversely, non-essential AAs (NEAAs) can be synthesized sufficiently in the body [43]. In this context, phenylalanine and leucine are classified as EAAs, while glutamic acid, arginine, glutathione, proline, and sarcosine are considered NEAAs. However, tyrosine is categorized as an NEAA as it is derived from phenylalanine [44]. CHA did not have significant effects on phenylalanine, leucine, or tyrosine, whereas it upregulated the levels of the above-mentioned NEAAs. In light of these findings, we illustrated the NEAA metabolic pathway by integrating transcriptomic and untargeted metabolomic data (Figure 5). It can be seen that UVB irradiation led to low expression of key genes (*Ggt1*, *Gsta3*, *Gstt3*, *Mgst1*, and *Oat*) associated with NEAA metabolism in mouse skin. Following the CHA intervention, the expression of these genes was upregulated. With the inclusion of three putatively annotated amino acids in the schematic network, i.e., aspartic acid, serine, and cystathionine, it can be inferred that CHA enhanced the NEAA metabolism in mouse skin.

### 3.7. CHA Treatment Modulated Polyunsaturated Fatty Acid Metabolism in the Skin of Photoaged Mouse Model

**Figure 6:**
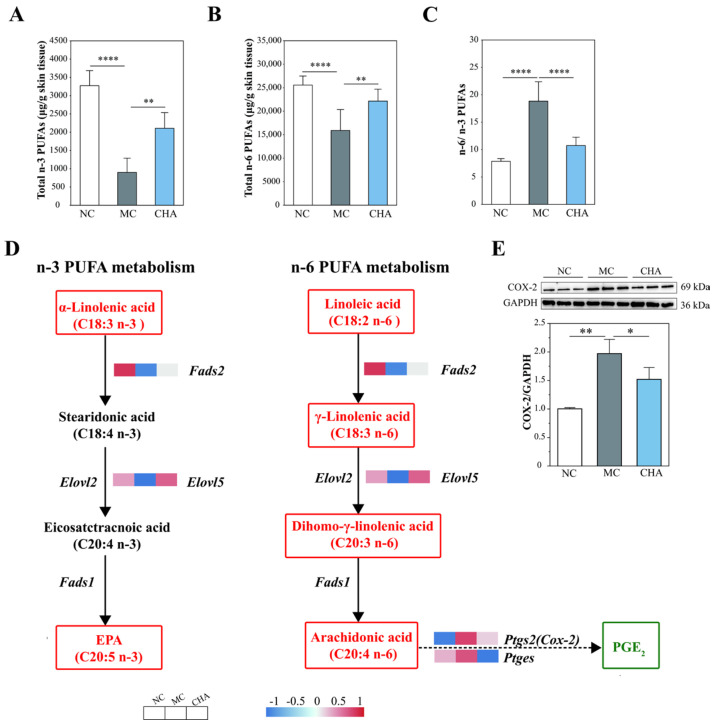
Effects of CHA on polyunsaturated fatty acid (PUFA) metabolism in the skin of a photoaged mouse model. (**A**) Total n-3 PUFAs levels in the mouse skin. (**B**) Total n-6 PUFAs levels in mouse skin. (**C**) Ratio of n-6/n-3 PUFAs in the mouse skin. For A-C, data are presented as mean ± SD (*n* = 6). (**D**) A schematic diagram of n-3/n-6 PUFA metabolic pathways annotated with multi-omics data. α-Linolenic acid, linoleic acid, γ-linolenic acid, and dihomo-γ-linolenic acid were significantly elevated following CHA treatment compared to the MC group, while the PGE_2_ level was decreased. There was no significant effect of CHA on levels of EPA and arachidonic acid compared to the MC group. The detailed identification of PGE2 can be seen in Table S3. (**E**) Western blot analysis of COX-2 protein expression in the mouse skin from NC, MC, and CHA groups. The results are normalized to GAPDH and shown as mean ± SD (*n* = 3). * *p* < 0.05, ** *p* < 0.01, **** *p* < 0.0001.

Among fatty acids, the polyunsaturated fatty acids (PUFAs), particularly those from n-6 and n-3 families, are recognized as crucial fatty acids for regulating immune and inflammatory responses. According to Table 1 and Figure 6A,B, CHA treatment elevated the levels of total n-6 and n-3 PUFAs, which were reduced following UVB exposure. Conversely, CHA treatment reduced the ratio of n-6/n-3 PUFAs toward basal levels, counteracting the increase induced by UVB irradiation (Figure 6C). Herein, the modulating effect of CHA on n-6 and n-3 PUFA metabolism was further validated by multi-omics integration (Figure 6D). Clearly, CHA treatment upregulated the expression of *Fads2* and *Elovl5*, genes involved in both n-3 and n-6 PUFA metabolism, and increased levels of typical n-3 and n-6 PUFAs, including α-linolenic acid (n-3), linoleic acid (n-6), γ-linolenic acid (n-6), and dihomo-γ-linolenic acid (n-6). Within the n-6 PUFA metabolism pathway, the conversion of arachidonic acid (AA) into PGE_2_ is a process primarily dependent upon the activity of COX-2. Western blot analysis indicated that exposure of mouse skin to UVB caused a strong expression of COX-2, while topical CHA application markedly attenuated this effect (Figure 6E).

## 4. Discussion

Despite being renowned for its antioxidant and anti-inflammatory properties [45,46], chamazulene (CHA) has not been thoroughly investigated for its potential applications in skin care. Our study demonstrates that topical application of 0.4% CHA effectively mitigated UVB-induced photoaging in mouse skin, as evidenced by improved skin structure and suppressed inflammation. Subsequent multi-omics analyses suggested that this protective effect may be mediated through the activation of PPARα/γ, leading to a comprehensive reprogramming of skin metabolism.

Previous studies have shown that photoaged human skin exhibits impaired fatty acid metabolism, characterized by downregulation of genes involved in fatty acid synthesis, such as *FADS1*, *FADS2*, *ELOVL3*, *ACC*, *FASN*, and *SCD* [8,13]. Intriguingly, our current study demonstrates that topical CHA application can reverse this metabolic defect in UVB-irradiated mouse skin. Bioinformatic analysis revealed the activating effects of CHA on genes associated with fatty acid metabolism, which encompass biosynthetic enzymes (*Accβ*, *Fasn*, *Elovl3*, *Elovl4*, *Elovl5*, *Elovl6*, *Fads2*, *Fads3*, *Fads6*, *Hacd2*, *Scd1*, and *Scd4*) and β-oxidation components (*Acox1*, *Acox2*, *Acox3*, *Acot12*, *Acot5*, *Acot6*, and *Acot7*). Prior studies have established that genetic or pharmacological inhibition of ACC, ELOVL, and SCD leads to impaired epidermal barrier function [47,48,49], resulting in pro-inflammatory cytokine production in the skin [50]. Fatty acid β-oxidation, primarily occurring in mitochondria and peroxisomes, is essential for cellular energy supply and also implicated in cellular senescence. In senescence and age-related degenerative diseases, there is a significant reduction in peroxisome β-oxidation [51]. While ACOX1, the rate-limiting enzyme that catalyzes peroxisomal β-oxidation of very-long-chain fatty acids, has proven anti-inflammatory and anti-aging properties [52]. As such, the substantial upregulation of the fatty acid metabolic pathway represents a key mechanism through which CHA treatment delays photoaging in mouse skin.

GC-MS analysis confirmed that CHA treatment significantly increased levels of bulk fatty acids in photoaged mouse skin, including predominant saturated, monounsaturated, and polyunsaturated types. These findings suggest that CHA may function as an agonist of PPARα/γ, thereby restoring fatty acid metabolism in photoaged skin. Interestingly, fatty acids themselves are endogenous ligands for PPARs in mammals [53]. It is plausible that a positive feedback regulation may be initiated upon CHA treatment: the initial activation of PPARα/γ could enhance fatty acid metabolism, and these newly synthesized fatty acids might in turn further activate PPARα/γ.

The p38 MAPK signaling cascade plays a key role in regulating UV-induced skin inflammation, as it triggers cytokine gene expression by means of transcriptional and posttranscriptional mechanisms [54]. In this study, UVB radiation did not elevate the synthesis of p38 MAPK protein in mouse skin; instead, it enhanced the activity of phospho-p38 MAPK. There is evidence suggesting that PPARα and PPARγ can modulate inflammatory responses by suppressing the activity of p38 MAPK in skin cells like keratinocytes and fibroblasts [23,55,56]. Therefore, our findings indicate that CHA may exert its anti-inflammatory effects, at least in part, through PPARα/γ-p38 MAPK signaling.

PUFAs play crucial roles in numerous inflammatory diseases. While n-6 PUFAs, especially AA and some of its eicosanoid products, contribute to inflammation, n-3 PUFAs like α-linolenic acid, EPA, and DHA exert anti-inflammatory effects [57]. This antagonistic effect is clinically significant, as higher ratios of n-6/n-3 PUFAs are strongly associated with skin inflammation [58,59]. Conversely, both systemic and topical supplementation with n-3 PUFAs have been shown to protect the skin from UV injury [60]. Therefore, we illustrated CHA’s effects on PUFA metabolism in photoaged mouse skin (Figure 6). Notably, CHA treatment upregulates key genes (*Fads2* and *Elovl5*) involved in both n-3 and n-6 PUFA metabolism and increases levels of typical n-3 and n-6 PUFAs (Figure 6A,B) while concurrently restoring the n-3/n-6 PUFA balance (Figure 6C).

In n-6 PUFA metabolism, eicosanoids derived from AA are frequently referred to in inflammatory diseases. Altered eicosanoid profiles emerge as a consequence of metabolic changes driven by the underlying inflammatory processes [61]. Mechanistically, UV radiation stimulates the release of AA from cell membrane phospholipids due to increased phospholipase activity [62,63]. Free AA is then metabolized into various eicosanoids under the action of cyclooxygenases (COXs), lipoxygenases (LOXs), or cytochrome P450 (CYPs 450) [61]. It has been shown that UV exposure leads to COX-2 overexpression in the skin, resulting in excessive production of prostaglandins, particularly PGE_2_, a well-known pro-inflammatory mediator [64,65]. In our study, UVB exposure reduced AA levels, possibly due to downregulated n-6 PUFA metabolism. Although CHA failed to modulate AA levels, potentially due to upregulation of the LOX and CYP450 pathways (Figure S6), it significantly attenuated UVB-induced COX-2 and PGE_2_ elevation. Since AA conversion to PGE_2_ is primarily dependent upon COX-2 activity, and p38 MAPK activation has been demonstrated to facilitate COX-2 expression in the UVB-irradiated skin [66,67], our data collectively suggest that CHA suppressed the PPARα/γ-p38MAPK-COX-2 axis in the photoaged mouse skin. Therefore, we provide the first evidence supporting a key role for PPARα/γ activation in the ameliorative effects of CHA on UVB-induced skin inflammation, which include restoring PUFAs homeostasis and inhibiting the p38 MAPK/COX-2 pathway.

In addition to fatty acids, CHA also induced amino acid reprogramming, another critical factor for maintaining skin health. The increase in phenylalanine, tyrosine, and leucine in UVB-exposed mouse skin may result from impaired protein biosynthesis [68] or, alternatively, reflect an adaptive response to UVB exposure by enhancing melanin production to absorb UVB rays [69,70]. Though CHA had no significant effect on these EAAs, it unexpectedly enhanced NEAA metabolism in the skin of photoaged mice. The elevated NEAAs include confidently identified arginine, sarcosine, glutathione, glutamic acid, and proline, as well as putatively identified serine and cystathionine. Emerging evidence shows that these NEAAs have great potential for alleviating skin aging or disorders, for instance, arginine promotes skin repair [71]. Furthermore, proline is one of the main substrates for collagen biosynthesis [72]; glutathione has antioxidant properties [73]; and serine is required for optimal keratinocyte growth [74]. Consequently, the increased NEAAs induced by CHA may act in combination with fatty acids to defend against UVB and promote skin structure remodeling. As we mentioned regarding the possible role of PPARα in amino acid metabolism in Section 3.5, additional work is warranted to determine whether PPARα activation directly increases the expression of genes involved in NEAA metabolism.

To our knowledge, this study is the first to delineate the molecular mechanisms of CHA through multi-omics approaches, elucidating its beneficial effects on skin health in the context of photoaging. Specifically, CHA restored fatty acid metabolism, particularly pro-/anti-inflammatory PUFAs and the downstream p38 MAPK/COX-2 pathway, and enhanced NEAA metabolism. Given its advantages of natural origin and lipophilic property, our findings render CHA a promising candidate for the treatment or prevention of photoaging. Although existing data support PPARα/γ as potential targets for combating photoaging, we cannot assume that the metabolic reprogramming induced by CHA treatment was completely in a PPARα/γ-dependent manner because of the lack of a PPARα/γ loss-of-function mouse model and specific antagonists. Nevertheless, we anticipate that our study will stimulate more research to explore the interaction between CHA and PPARα/PPARγ, thereby solidifying CHA’s favorable effects on the skin.

## Figures and Tables

**Table 1:** Quantification of polyunsaturated fatty acids in mouse skin (*n* = 6).

Metabolites	Abbreviation	RT (min)	Concentration (μg/g Skin Tissue)		
			NC	MC	CHA
Linolelaidic acid	C18:2n-6	14.56	20.04 ± 3.96 a	8.47 ± 1.50 b	15.39 ± 1.70 a
Linoleic acid	C18:2n-6 (LA)	14.90	24,308.99 ± 1867.58 a	15,052.33 ± 4389.97 b	21,228.26 ± 2533.12 a
γ-Linolenic acid	C18:3n-6	15.23	113.00 ± 16.37 a	36.90 ± 6.70 b	83.13 ± 27.85 a
α-Linolenic acid	C18:3n-3 (ALA)	15.62	3026.82 ± 410.89 a	663.88 ± 162.19 c	1899. 28 ± 180.36 b
11Z,14Z-Eicosadienoic acid	C20:2n-6	17.38	225.62 ± 36.75 a	111.60 ± 13.30 c	174.74 ± 16.03 b
Dihomo-γ-linolenic acid	C20:3n-6 (DGLA)	17.77	119.20 ± 17.33 a	45.29 ± 12.27 c	71.58 ± 5.56 b
11Z,14Z,17Z-Eicosatrienoic acid	C20:3n-3	18.21	14.00 ± 1.96 a	5.10 ± 0.72 c	9.48 ± 0.61 b
Arachidonic acid	C20:4n-6 (AA)	18.02	714.07 ± 100.62 a	573.43 ± 72.77 b	532.03 ± 63.57 b
5Z,8Z,11Z,14Z,17Z-Eicosapentaenoic acid	C20:5n-3 (EPA)	18.876	16.54 ± 3.21 a	5.86 ± 2.10 b	9.43 ± 2.36 b
13Z,16Z-Docosadienoic acid	C22:2n-6	20.06	3.30 ± 1.06 a	2.70 ± 0.59 b	6.04 ± 0.93 b
Adrenic acid	C22:4n-6	20.87	43.71 ± 3.28 a	47.45 ± 9.67 a	39.69 ± 5.01 a
7Z,10Z,13Z,16Z,19Z-Docosapentaenoic acid	C22:5n-3 (DPA)	21.88	47.03 ± 4.33 a	42.76 ± 6.76 a	39.23 ± 9.04 a
4Z,7Z,10Z,13Z,16Z,19Z-Docosahexaenoic acid	C22:6n-3 (DHA)	22.13	186.56 ± 23.03 a	197.88 ± 32.88 a	162.90 ± 40.55 a
Total			28,838.89 ± 2315.94 a	16,973.63 ± 4806.88 b	24,271.17 ± 2908.76 a

RT: Retention time. Values with different letters indicate significant differences (*p* < 0.05).

## Data Availability

The raw transcriptomic sequencing data are being deposited in the NCBI database and will be available upon acceptance of the manuscript. All other data supporting the findings of this study are available from the corresponding author upon reasonable request.

## Supplementary Materials

The following supporting information can be downloaded at https://www.mdpi.com/article/10.3390/antiox14111320/s1: Figure S1: Macroscopic appearance of the 0.4% (*w*/*w*) CHA emulsion. Figure S2: The infiltration of inflammatory cells in mouse skin by H&E staining (100×). Figure S3: Principal component analysis (PCA) score plot of skin transcriptome data from NC, MC, and CHA groups. Figure S4: KEGG pathway map for IL-17 signaling pathway. Figure S5: Validation of OPLS-DA models by 200 permutation tests. Figure S6: DEGs in the CYP450 and LOX pathway of arachidonic acid metabolism. Table S1: Sequences of the primers for qRT-PCR. Table S2: Quantification of saturated and monounsaturated fatty acids in mouse skin (n = 6). Table S3: Mass spectrometry data of key DEMs. Table S4. Calibration data for the quantitative analysis of fatty acids by GC-MS.

## Abbreviations

The following abbreviations are used in this manuscript:

AEO	*Artemisia sieversiana* Ehrhart ex Willd. Essential oil
AA	Arachidonic acid
CHA	Chamazulene
CCL	CC ligand
CXCL	CXC chemokine ligand
COX-2	Cyclooxygenase-2
DEGs	Differentially expressed genes
ECM	Extracellular matrix
FAME	Fatty acid methyl ester
GC-MS	Gas chromatography–mass spectrometry
GSEA	Gene set enrichment analysis
HYP	Hydroxyproline
IL	Interleukin
MAPK	Mitogen-activated protein kinase
MMP	Matrix metalloproteinase
NEAA	Non-essential amino acid
OPLS-DA	Orthogonal partial least squares discriminant analysis
PCA	Principal component analysis
PUFA	Polyunsaturated fatty acid
PPAR	Peroxisome proliferator-activated receptor
UPLC-MS/MS	Ultra-performance liquid chromatography–tandem mass spectrometry
