# Supplementary material for: Chamazulene Induces Metabolic Reprogramming and Mitigates Inflammation in Photoaged Skin: PPARα/γ as Potential Regulators
Source: Antioxidants (Basel). 2025 Oct 31;14(11):1320. doi: 10.3390/antiox14111320 (PMC12649271; doi:10.3390/antiox14111320)
Supplement: Supplementary file 1 [file antioxidants-14-01320-s001.zip › antioxidants-3915914-supplementary.pdf]

Supporting information

Chamazulene induces metabolic reprogramming and mitigates inflammation in photoaged skin: PPARα/γ as potential regulators

Ying Zhou [1,2], Wencui Wang [2], Lei He [3], Nan Zhang [2], Bowen Zhou [2], Zimeng Chen [2], Li Ma [2,*], Lei Yao [2,*]

[1] Department of Resources and Environment, School of Agriculture and Biology, Shanghai Jiao Tong University, 800 Dongchuan Road, Shanghai, 200240, China; zhouyingmap@sjtu.edu.cn

[2] Research and Development Center of Aromatic Plants, School of Design, Shanghai Jiao Tong University, 800 Dongchuan Road, Shanghai, 200240, China; zhouyingmap@sjtu.edu.cn; wwh123@sjtu.edu.cn; fxzwzhangnan@sjtu.edu.cn; zhou.bw@sjtu.edu.cn; zimengchen@sjtu.edu.cn; malimali2006@sjtu.edu.cn; yaolei@sjtu.edu.cn

[3] School of Public Health, Hongqiao International Institute of Medicine, Shanghai Jiao Tong University School of Medicine, 227 South Chongqing Road, Shanghai, 200025, China; 018150910007@sjtu.edu.cn

* Correspondence: yaolei@sjtu.edu.cn (L. Y.); malimali2006@sjtu.edu.cn (L. M.);

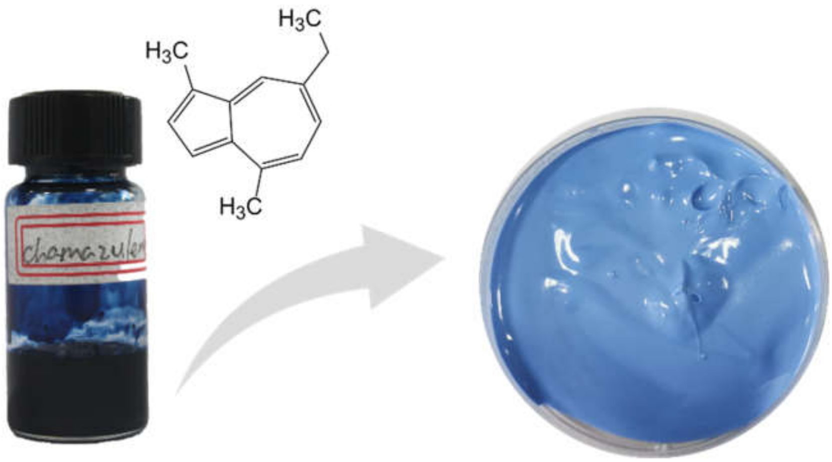

Figure S1. Macroscopic appearance of the 0.4% (w/w) CHA emulsion.

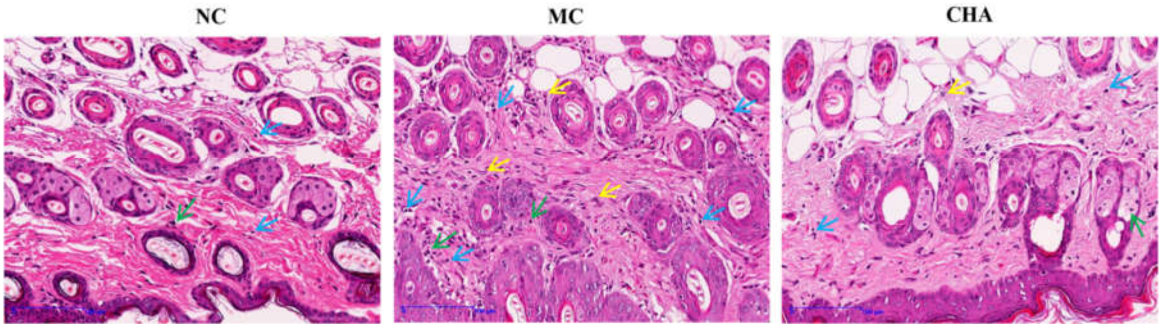

Figure S2. The infiltration of inflammatory cells in mouse skin by H&E staining (100 ×). The bule, green yellow arrows show the lymphocytes, neutrophils, monocytes, respectively. Scale bars = 100 μm.

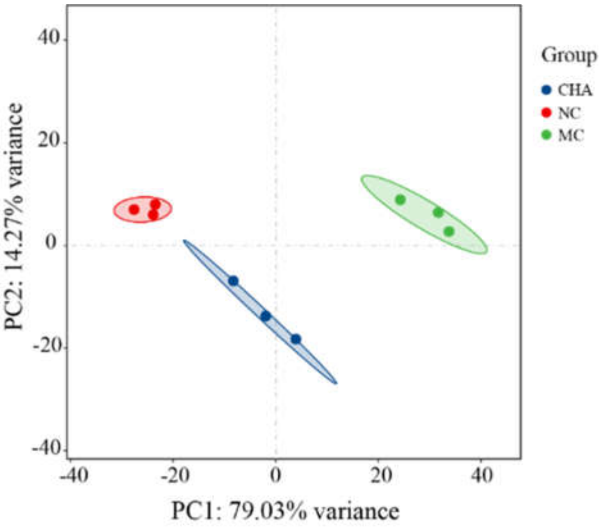

Figure S3. Principal component analysis (PCA) score plot of skin transcriptome data from NC, MC, and CHA groups. Each point represents an individual sample.

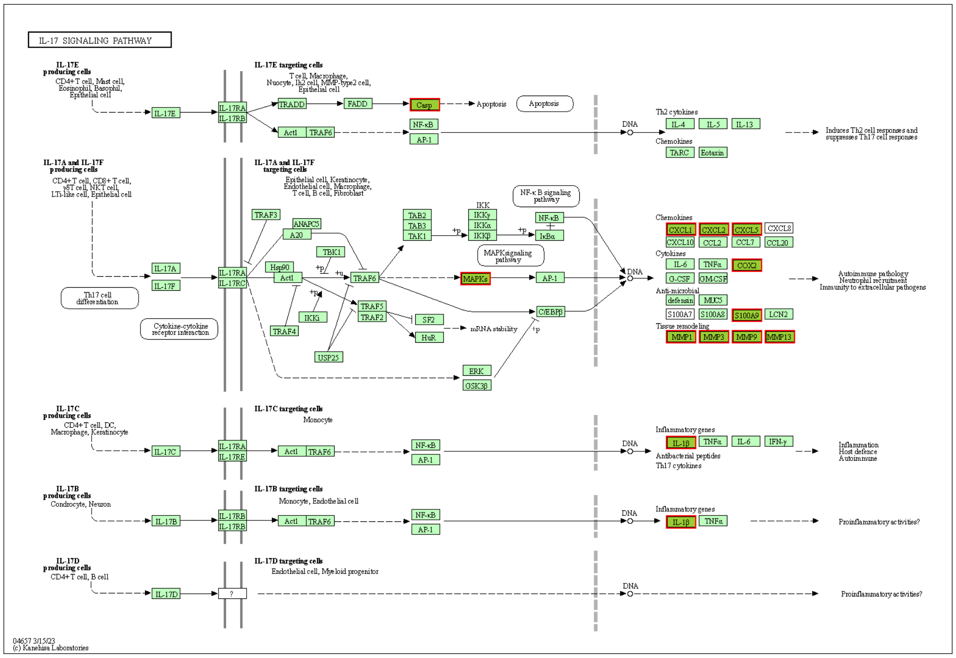

Figure S4. KEGG pathway map for IL-17 signaling pathway. Red boxes indicate genes that are significantly downregulated in the mouse skin of NC and CHA-treated groups compared to the MC group.

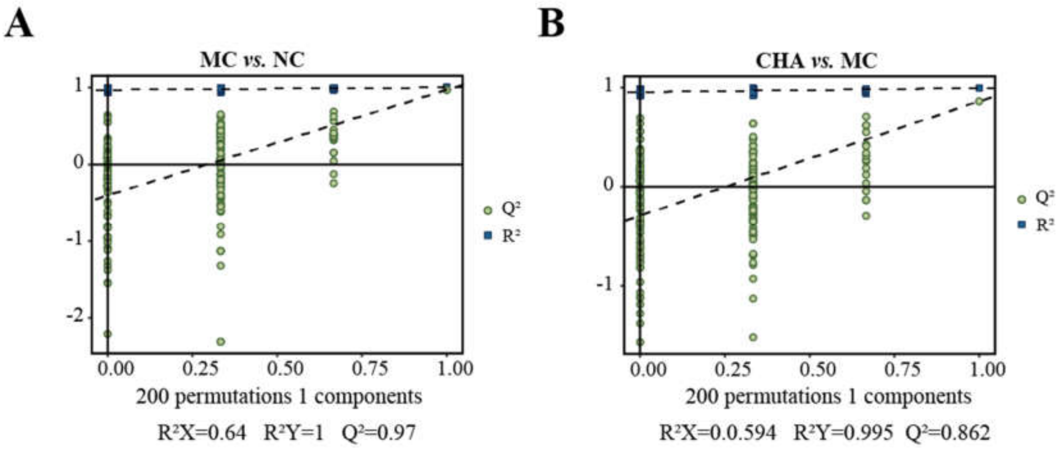

Figure S5. Validation of OPLS-DA models by 200 permutation tests.

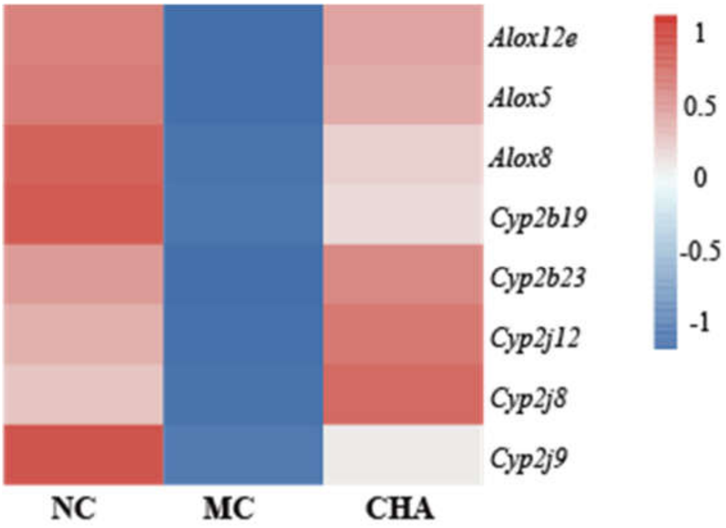

Figure S6. DEGs in the CYP450 and LOX pathway of arachidonic acid metabolism.

Table S1. Sequences of the primers for qRT-PCR

Genes	Sequences	Product Size (bp)
β-actin	Forward: 5′-TATGCTCTCCCTCACGCCATCC-3′<br>Reverse: 5′-GTCACGCACGATTTCCCTCTCAG-3′	129
Il1β	Forward: 5′-TCGCAGCAGCACATCAACAAGAG-3′<br>Reverse: 5′-TCCACCACCCTGTTGCTGTAG-3′	97
Mmp9	Forward: 5′-CGCCACCACAGCCAACTATGAC-3′<br>Reverse: 5′-CTGCTTGCCCAGGAAGACGAAG-3′	130
Cxcl2	Forward: 5′-CACTGGTCCTGCTGCTGCTG-3′<br>Reverse: 5′-GCGTCACACTCAAGCTCTGGATG-3′	135
CCl3	Forward: 5′-CTCCCAGCCAGGTGTCATTTTCC-3′<br>Reverse: 5′-CAGGCATTCAGTTCCAGGTCAGTG-3′	108
Pank1	Forward: 5′-GCAGTGTACTCCAAGGACAACTAC-3′<br>Reverse: 5′-AACCAGTCAGCAAGCAACATAGG-3′	91
Fads2	Forward: 5′-TTCAGCGGGCACCTCAATTTCC-3′<br>Reverse: 5′-ACGGCTTCTCCTGGTATTCAATGC-3′	133
Elovl5	Forward: 5′-CCACCACGCTACCATGCTCAAC-3′<br>Reverse: 5′-GGACGGGATGGAGGACAGACC-3′	139
Scd3	Forward: 5′-ACGACCACCACCACCATCAC-3′<br>Reverse: 5′- TTCATTTCAGGACGGATGTCTTCTTC-3′	110

Table S2. Quantification of saturated and monounsaturated fatty acids in mouse skin (n=6)

Metabolites	Abbreviation	RT (min)	Concentration (μg/g skin tissue)		
			NC	MC	CHA
			Saturated fatty acids (SFAs)		
Hexanoic acid	C6:0	5.89	0.28 ± 0.08 a	0.22 ± 0.01 a	0.27 ± 0.02 a
Octanoic acid	C8:0	6.97	1.31 ± 0.58 a	0.93 ± 0.20 a	1.46 ± 0.46 a
Decanoic acid	C10:0	8.01	10.59 ± 2.93 a	3.01 ± 0.65 b	6.87 ± 0.94 c
Undecanoic acid	C11:0	8.53	0.54 ± 0.03 a	0.20 ± 0.06 b	0.41 ± 0.06 c
Lauric acid	C12:0	9.07	76.54 ± 14.63 a	42.39 ± 7.02 b	78.10 ± 18.54 a
Tridecanoic acid	C13:0	9.68	2.98 ± 0.30 a	1.53 ± 0.23 b	2.74 ± 0.47 a
**Myristic acid**	**C14:0**	**10.34**	**1292.83 ± 185.06 a**	**679.41 ± 112.41 b**	**1155.56 ± 244.02 a**
Pentadecanoic acid	C15:0	11.10	243.33 ± 31.43 a	103.54 ± 17.77 b	207.74 ± 38.94 a
**Palmitic acid**	**C16:0**	**11.97**	**11339.10 ± 845.04 a**	**7621.36 ± 853.84 b**	**10167.68 ± 983.90 a**
Heptadecanoic acid	C17:0	12.91	273.58 ± 51.38 a	139.56 ± 21.90 b	266.85 ± 41.75 a
Stearic acid	C18:0	14.00	2886.09 ± 418.98 a	2506.16 ± 581.66 a	3139.58 ± 315.38 a
Arachidic acid	C20:0	16.37	714.08 ± 100.61 a	573.43 ± 72.77 a	532.04 ± 63.57 a
Heneicosanoic acid	C21:0	17.65	38.15 ± 7.15 a	3.63 ± 0.41 b	43.53 ± 6.15 a
Behenic acid	C22:0	18.96	48.82 ± 5.30 a	24.85 ± 6.47 b	84.40 ± 16.28 c
Tricosanoic acid	C23:0	20.30	11.80 ± 2.31 a	3.78 ± 0.48 b	15.73 ± 1.76 a
Lignoceric acid	C24:0	21.77	38.51 ± 6.62 a	23.82 ± 2.71 b	45.61 ± 8.22 a

Metabolites	Abbreviation	RT (min)	Concentration (μg/g skin tissue)		
			NC	MC	CHA
Total			16418.16 ± 1501.79 a	11211.29 ± 3066.23 b	15385.86 ± 1474.03 a
				**Monounsaturated fatty acids (MUFAs)**	
Myristoleic acid	C14:1n-5	10.65	125.46 ± 31.58 a	39.11 ± 8.55 b	78.11 ± 16.05 b
Palmitoleic acid	C16:1n-7	12.27	7647.43 ± 652.79 a	3505.93 ± 932.65 b	5949.89 ±621.97 a
10Z-Heptadecenoic acid	C17:1n-7	13.22	253.62 ± 33.61a	80.46 ± 16.84 b	196.34 ± 20.33 a
Oleic acid	C18:1n-9	14.32	18995.43 ± 1377.05 a	13615.81 ± 3341.61 b	17184.96 ± 1883.22 a
11Z-Eicosenoic acid	C20:1n-9	16.72	782.78 ± 49.09 a	341.01 ± 43.21 b	595.51 ± 61.08 c
Erucic acid	C22:1n-9	19.34	88.97 ± 10.88 c	19.16 ± 1.78 b	151.80 ± 20.99 a
Nervonic acid	C24:1n-9	22.26	6.21 ± 0.84 b	3.37 ± 0.63 c	10.88 ± 1.63 a
Total			27961.28 ± 2073.73 a	17637. 02 ± 5120.95 b	24215.91 ± 3393.73 a

RT: Retention time. Values with different letters indicate significant differences ($p < 0.05$).

Table S3 Mass spectrometry data of key DEMs

No.	RT (min)	Ion mode	Metabolites	m/z	Molecular formula	Fragmentation score	Mass Error (ppm)	Main fragments (MS/MS)	Level
1	0.64	Pos	Arginine	175.119	$C_6H_{14}N_4O_2$	56	-1.78456	271.21; 315.20; 333.21; 189.13; 233.12	Level 1
2	0.70	Pos	Glutamic acid	148.06	$C_5H_9NO_4$	87.5	-2.18192	84.04; 102.05; 130.05; 148.06	Level 1
3	0.70	Pos	Sarcosine	90.0548	$C_3H_7NO_2$	45.3	-1.98736	90.05; 72.08; 73.05	Level 1
4	0.74	Pos	Proline	116.07	$C_5H_9NO_2$	93.8	-1.92809	70.07; 116.07; 98.98	Level 1
5	0.83	Neg	Glutathione	306.076	$C_{10}H_{17}N_3O_6S$	90.9	-0.16431	306.08; 143.05; 272.09; 128.03; 102.96	Level 1
6	1.09	Neg	N-Acetyl-L-aspartic acid	174.04	$C_6H_9NO_5$	76.1	-5.56731	88.04; 130.05; 58.03; 174.04	Level 1
7	1.13	Neg	Tyrosine	180.066	$C_9H_{11}NO_3$	97.4	-4.90049	180.07; 163.04; 119.05	Level 1
8	1.42	Pos	Leucine	132.102	$C_6H_{13}NO_2$	85.3	-1.38887	86.10; 72.94; 113.96	Level 1
9	2.24	Neg	Phenylalanine	164.071	$C_9H_{11}NO_2$	95.3	-6.22796	147.04; 164.08; 72.01	Level 1
10	7.49	Neg	PGE_2	351.218	$C_{20}H_{32}O_5$	97.6	-0.18741	271.21; 315.20; 333.20; 189.13; 233.12	Level 1

RT: Retention time.

Table S4. Calibration data for the quantitative analysis of fatty acids by GC-MS

No.	Metabolites	Retention time (min)	Standard curve	R^2	Linear range (ug/mL)
1	Hexanoic acid	5.89	y = 1.119190x-6.395186E-005	0.9999	0.002-50
2	Octanoic acid	6.97	y = 2.320197x-2.918181E-004	1.0000	0.002-50
3	Decanoic acid	8.01	y = 1.722523x-2.326997E-005	0.9999	0.004-100
4	Undecanoic acid	8.53	y = 1.691269x-7.461453E-006	1.0000	0.002-50
5	Lauric acid	9.07	y = 1.597473x-9.983890E-005	1.0000	0.004-100
6	Tridecanoic acid	9.68	y = 1.663789x -3.496303E-005	1.0000	0.002-50
7	Myristic acid	10.34	y = 1.592229x+6.849841E-004	1.0000	0.002-50
8	Myristoleic acid	10.65	y = 0.369258x-4.675281E-005	1.0000	0.002-50
9	Pentadecanoic acid	11.10	y = 1.483835x-9.215995E-005	1.0000	0.002-50
10	Palmitic acid	11.97	y = 1.570627x+0.006935	1.0000	0.004-100
11	Palmitoleic acid	12.27	y = 0.306691x+5.124348E-004	1.0000	0.002-50
12	Heptadecanoic acid	12.91	y = 1.468932x+6.819757E-004	1.0000	0.004-100
13	10Z-Heptadecenoic acid	13.22	y = 0.320659x+2.717622E-004	1.0000	0.002-50
14	Stearic acid	14.00	y = 1.481514x+0.004857	1.0000	0.004-100
15	Oleic acid	14.32	y = 0.302666x+9.742029E-004	1.0000	0.004-100
16	Linolelaidic acid	14.56	y = 0.398997x+3.870560E-005	1.0000	0.002-50
17	Linoleic acid	14.90	y = 0.409144x+9.443471E-004	1.0000	0.002-50
18	γ-Linolenic acid	15.23	y = 0.362778x+1.161557E-004	1.0000	0.004-100

No.	Metabolites	Retention time (min)	Standard curve	R^2	Linear range (ug/mL)
19	α-Linolenic acid	15.62	y = 0.468845x+2.705423E-004	1.0000	0.002-50
20	Arachidic acid	16.37	y = 1.307506x+3.551766E-004	1.0000	0.004-100
21	11Z-Eicosenoic acid	16.72	y = 0.305217x+5.223376E-006	1.0000	0.002-50
22	11Z,14Z-Eicosadienoic acid	17.38	y = 0.373994x -6.972350E-007	1.0000	0.002-50
23	Dihomo-γ-linolenic acid	17.77	y = 0.334236x+4.775550E-005	1.0000	0.002-50
24	11Z,14Z,17Z-Eicosatrienoic acid	18.21	y = 0.475223x-2.503628E-005	1.0000	0.002-50
25	Arachidonic acid	18.02	y = 0.360534x+7.224993E-006	1.0000	0.002-50
26	5Z,8Z,11Z,14Z,17Z-Eicosapentaenoic acid	18.876	y = 0.372092x-3.520331E-005	0.9997	0.002-50
27	Heneicosanoic acid	17.65	y = 1.232484x+4.311022E-004	1.0000	0.002-50
28	Behenic acid	18.96	y = 1.229369x+9.585484E-005	1.0000	0.004-100
29	Erucic acid	19.34	y = 0.306434x+1.773694E-005	1.0000	0.002-50
30	13Z,16Z-Docosadienoic acid	20.06	y = 0.352240x+2.757960E-005	1.0000	0.002-50
31	Adrenic acid	20.87	y = 0.348707x+1.106857E-006	1.0000	0.002-50
32	7Z,10Z,13Z,16Z,19Z-Docosapentaenoic acid	21.88	y = 0.391718x+8.116770E-005	1.0000	0.002-50
33	4Z,7Z,10Z,13Z,16Z,19Z-Docosahexaenoic acid	22.13	y = 0.368379x+1.414388E-004	1.0000	0.002-50
34	Tricosanoic acid	20.30	y = 1.115295x+3.086098E-005	1.0000	0.002-50
35	Lignoceric acid	21.77	y = 1.088535x+2.962989E-004	1.0000	0.004-100
36	Nervonic acid	22.26	y = 0.300435x+4.097607E-004	1.0000	0.002-50
